# Opioid Pain Medication Use in Athletes

**DOI:** 10.7759/cureus.115735

**Published:** 2026-09-03

**Authors:** LaRae L Seemann, Rosa M Pasculli, Oluseun A Olufade, Shanterian King, Jason Lee, Mohit Chauhan, Amr R Saleh, George G. A Pujalte

**Affiliations:** 1 Department of Family Medicine and Orthopedics and Sports Medicine, Mayo Clinic in Florida, Jacksonville, USA; 2 Department of Orthopedics, Emory University School of Medicine, Atlanta, USA; 3 Department of Physical Medicine and Rehabilitation and Sports Medicine, Mayo Clinic in Florida, Jacksonville, USA; 4 Department of Sports Medicine, Hudson Physicians, Hudson, USA; 5 Department of Psychiatry and Psychology, Mayo Clinic in Florida, Jacksonville, USA

**Keywords:** analgesics, athletes, narcotics, opioid, prescription drug misuse

## Abstract

The use of opioids for pain management has become increasingly accepted over the years. There are known adverse effects of opioids that have implications for athletes when used for pain relief in the context of sports. Our objective was to conduct a review of the available literature to evaluate the following: (1) opioid use in athletes; (2) how to prevent initial use; (3) tapering opioids; and (4) nonopioid alternatives. A literature review was conducted on the prevalence of opioid use in athletes, strategies for preventing initial use and tapering opioids, and nonopioid alternatives for pain management in athletes. Use of opioids early in an athlete’s career appears to increase the risk of subsequent use and misuse at college and professional levels. Prevention of initial use of opioids, establishing a timeline outlining the course and expectations for opioid use, and understanding the individual’s level of sport and specific involvement are all important factors that can help prevent opioid misuse. More research is needed for clinicians to understand the level of use and misuse of narcotic pain medications in the athletic and active population. This issue should receive more consideration as the level of play increases. There are indications that opioid use has now become as prevalent in high school athletes as in higher levels of play, a trend that should be monitored and possibly curtailed in recognition of the greater risks in adolescents when it comes to narcotic pain medications.

## Introduction and background

In the decades leading up to the 1990s, the medical community had generally avoided opioids as a treatment option for pain in what some authors referred to as a period of opiophobia [1]. Multiple publications during the 1980s and early 1990s questioned the undertreatment of pain and recommended opioids as a treatment option for various conditions, including cancer-related and chronic pain [2-4].

In the years following, there was a national effort to address the perceived underassessment and undertreatment of pain. The Joint Commission on Accreditation of Healthcare Organizations (Joint Commission) published new recommendations for pain management in 2000, which was followed by statements from the Federation of State Medical Boards and the Drug Enforcement Administration assuring opioid prescribers of less oversight and regulation [5-7]. These changes, combined with increased marketing by pharmaceutical companies and newer drug formulations misrepresented to physicians as having a lower risk of misuse, resulted in drastically increased prescription opioid consumption [8-9].

Updated pain management standards from The Joint Commission were published in 2017, and measures to reduce opioid prescribing have been introduced, with a resulting decrease in prescriptions and opioid-related deaths [10-14]. These measures include prescription drug monitoring programs, the National All Schedules Prescription Electronic Reporting Act, and required continuing education for all opioid-prescribing clinicians [13-15]. Most opioid medications are classified as Schedule II controlled substances given the potential for misuse and addiction; they require a written prescription by a licensed practitioner, and refills are prohibited.

Opioids produce an analgesic effect by binding to opioid μ-receptors; however, this can also cause drowsiness or euphoria, known as opioid-induced euphoria [16]. With chronic use of opioids, tolerance can develop and manifest as a shorter duration of pain relief, less sedation, or less euphoria. This will require the user to either decrease the interval between doses or increase the dose to achieve the same effects, which then increases the risks of other adverse effects, such as respiratory depression [17]. Physical dependence will result in withdrawal symptoms if the drug is acutely discontinued or the patient is administered an opioid antagonist. The CDC defines opioid misuse as use of a prescription in a manner other than as directed by a physician, for example, in greater amounts, more often, longer than instructed, or by using someone else’s prescription [18].

Athletes are at risk for musculoskeletal and non-musculoskeletal injuries that may lead to acute and chronic pain. These injuries may necessitate complex treatments, including surgical interventions and long recovery periods. In the management of acute and postoperative pain, opioids are sometimes prescribed due to their potent analgesic effects. Acute injuries like fractures, dislocations, and ligament tears can result in intense pain that may require opioids in the short term [19]. Following surgical procedures such as arthroscopic surgery for joint repair or reconstruction (e.g., anterior cruciate ligament tears), opioids are often used to manage postoperative pain. Opioid use in sports medicine raises significant concerns, primarily due to their potential for addiction, impaired performance, and long-term health risks [16,20].

However, surrounding pressures from teammates, parents, coaches, and fans to win; pressure to specialize in their sport; and a variety of psychosocial pressures can lead the athlete to ignore the pain and play through it [19,20]. Pain medications such as prescription opioids offer a vehicle for athletes to mask their symptoms and remain in their sport. An increasing number of studies show that opioid use and misuse are a concern among athletes of all levels, especially in adolescents [21-23]. The purpose of this review is to explore current evidence on opioid use in athletes and discuss strategies for prevention, tapering, and opioid-sparing options currently in practice. We will explore the risk factors for prescription opioid use in athletes, including the prevalence in specific sport populations such as high school athletes and professional soccer and football players, with a brief overview of alternative pain medications.

## Review

Methods

Review Design and Literature Search

This study was conducted as a narrative review of the literature examining opioid use and pain management among athletes. The review focused on four principal areas: (1) the prevalence and patterns of opioid use and misuse among athletes; (2) strategies for preventing initial opioid use; (3) approaches to opioid tapering; and (4) nonopioid alternatives for pain management.

A literature search was conducted using PubMed, Scopus, and Google Scholar for publications available from January 1, 1985, through December 31, 2021. The final search was performed on June 20, 2023. Search concepts included terms related to athletes and sports participation, opioids and opioid prescribing, sports-related injuries, and pain management. The search strategy combined these concepts using Boolean operators. The principal search strategy was (athlete OR sport OR "sports medicine" OR "sports-related injury") AND (opioid* OR narcotic* OR "opioid analgesic*" OR "prescription opioid*" OR "opioid prescription*") AND (pain OR "pain management" OR injur* OR analges*).

Additional targeted searches were performed using combinations of the terms "opioid use in athletes", "sports-related injuries", "pain management in sports", and "opioid prescriptions in sports medicine" to identify literature relevant to the major topics of the review. Reference lists of relevant articles and reviews were also examined to identify additional publications.

Eligibility and Study Selection

Publications were considered eligible if they were written in English and addressed opioid use, opioid prescribing, opioid misuse, pain management, opioid tapering, or alternatives to opioids in athletes or sports-related clinical settings. Original research articles, relevant review articles, and clinical guidelines were considered. Publications focused exclusively on pain management unrelated to sports or athletic populations and publications not available in English were excluded.

Records identified through the database searches were initially assessed for relevance based on their titles and abstracts. Potentially relevant publications were then subjected to full-text review to determine whether they addressed one or more of the prespecified topics of interest. Two authors independently screened the articles. Disagreements regarding eligibility were resolved through discussion until consensus was reached. A total of 32 publications meeting the review criteria were included in the qualitative synthesis.

Data Synthesis and Quality Assessment

Because the objective of this article was to provide a narrative overview rather than conduct a systematic review or meta-analysis, the included literature was synthesized qualitatively. Findings were organized according to the principal clinical themes of opioid use among high school and collegiate athletes, opioid use among professional athletes, prevention of initial opioid use, opioid tapering, and alternatives to opioids.

A formal study-level risk-of-bias or methodological quality assessment using a standardized systematic-review instrument was not performed. This should be considered when interpreting the review's conclusions, as the included literature encompasses different study designs and levels of evidence. Figure 1 shows the flow diagram of the study selection.

**Figure 1:**
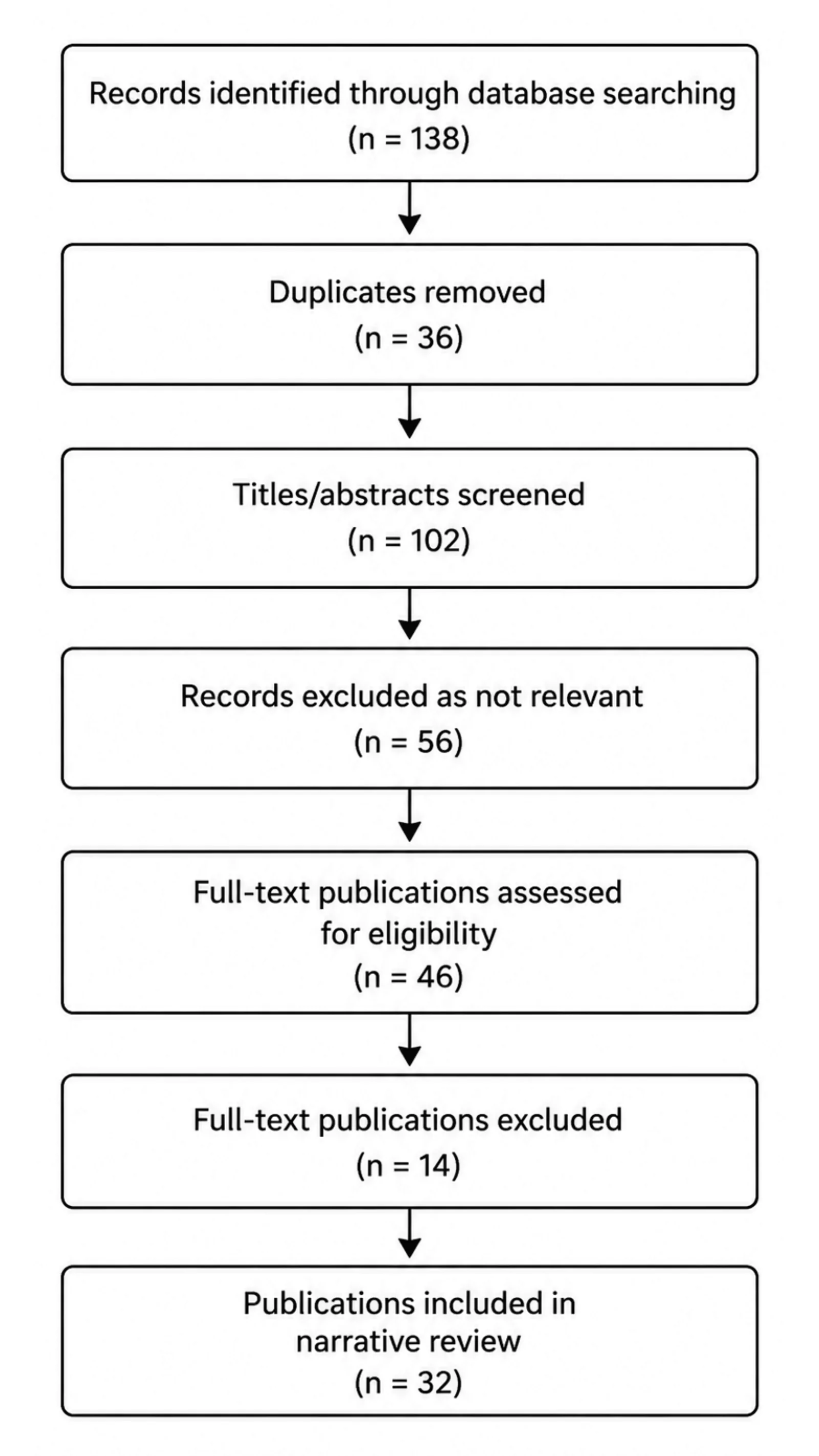
Flow diagram of the study selection

A total of 32 publications were included in the review synthesis. An additional 13 references were cited to provide background and contextual information, bringing the total to 45 in the manuscript.

Understanding the issue

High School and Collegiate Athletes

It is estimated that 28.4% to 46.4% of high school athletes have used opioids in their lifetime, and use of opioids early in an athlete’s career appears to increase the risk of subsequent use and misuse at college and professional levels. This wide range reflects variability in literature samples, including primary studies of various designs and different sport types (contact and non-contact), as well as reliance on self-report in some studies [24,25]. Although there is evidence to suggest that adolescent involvement in sports could serve as a protective factor against lifetime heroin use, the high rate of painful injuries that require medical attention may put young athletes at risk of opioid misuse, and research examining this relationship as it relates to opioid misuse in high school athletes is limited [26]. Unfortunately, research related to the prevalence of opioid use in athletes is limited, with specific data on misuse being insufficient, likely secondary to the difficulty in detecting misuse.

Among high school athletes, factors that increase the likelihood of nonmedical opioid use include the type of sport, history of injury, male sex, and playing at the varsity level [24,26,27]. Athletes participating in contact sports (e.g., wrestling, football, ice hockey) and male adolescents involved in organized sports seem to be at greatest risk for using and misusing opioids [24,28]. There are approximately 7.5 million adolescents in the US playing high school sports, with approximately 2 million athletic injuries recorded in this group each year. With 2 million recorded injuries, the potential for prescription opioid management is high, though data suggest not all groups are at similar risk for use and misuse. A study of 1,540 high school students revealed that male athletes used or misused opioids at least once a year more than male nonathletes. However, when comparing female athletes to female nonathletes, the risk was similar between the groups. Many factors play a role in adolescent athletes’ ability to obtain opioids, including increased opportunity for misuse by acquiring them from a teammate who is diverting opioid medication to their peers [29,30]. This pattern among young athletes was reinforced by another review, which emphasized that adolescent athletes require prescribing guidance tailored to their developmental stage, rather than extrapolating from general-population recommendations for adults [31].

As high school athletes transition to collegiate athletics, the risk of injury increases. Approximately 460,000 National Collegiate Athletic Association (NCAA) athletes compete across 24 sports on an annual basis. Kay et al. analyzed 3,183 recorded injuries during the 2009-2010 to 2014-2015 seasons. About 30% of those injuries required surgical intervention and were likely prescribed at least a short course of opioids [32]. A 2013 national study specifically analyzing substance use in male NCAA athletes found that almost 2,000 reported taking prescription narcotics for either medical or recreational purposes [33].

Professional Athletes

Surprisingly, the cumulative opioid use in high school athletes is similar to that of professional athletes. This is a disconcerting discovery, especially given the increasing pressure for early sports specialization and the high rates of overuse injuries. Given the potential for early opioid prescription and misuse in young athletes to serve as a pipeline for later dependence, it comes as no surprise that patterns of opioid use or misuse in amateur athletes are often maintained as athletes progress in their careers [26]. Opioid use in professional athletes at any given time ranges from 4% to 5% based on studies evaluating use in Fédération Internationale de Football Association (FIFA) futsal athletes and professional dancers [24,34,35]. Though these numbers seem less concerning, the percentage is higher for certain sports and cumulatively over a sporting career. There are limited data available on cumulative use of opioids in most professional sports; the National Football League (NFL) is the most extensively studied professional sports league. In a 2011 study, the reported proportion of athletes who used opioids during a typical NFL career was a staggering 52% [36].

Research has revealed that 71% of former professional football players misused prescription opioids during their careers, and current opioid misuse among NFL retirees is over four times that of the general population (i.e., 7% vs. 1.6%) [36]. Furthermore, when specifically considering NFL retirees, opioid use in early retirement was a strong predictor of continued use [36,37]. Those athletes with continued opioid use were more likely to experience considerable negative effects on their mental health, for example, diagnoses of moderate to severe depression over the subsequent decade or exhibiting symptoms consistent with a diagnosis of depression [37]. The deleterious effects of opioid use on retired athletes are perhaps most concerning, as it raises the question of whether there is a summative effect of opioid use on the athlete over their lifespan. If so, intervention in youth sports may help prevent later health issues for athletes.

Preventing initial use

The decision to manage an athlete’s pain with opioids should be carefully weighed. Factors to consider in this decision-making process include the severity of the athlete’s pain, the safety of medication use, the return-to-play plan, prescribing regulations, and the level at which the athlete is competing [38]. Persistent pain can accompany the athlete throughout their career and beyond, thus potentially prolonging opioid use [39]. One study among retired NFL players showed only 14.3% of players used opioids as prescribed, while 11.9% misused opioids [40].

Opioid use is a particular concern among athletes because of their high risk of injury and the pressure to perform. There are high rates of misuse, with risk building progressively, starting at the high school level [24]. Participation in contact sports seems to be the most significant predictor of opioid use and misuse, with sports like weightlifting and wrestling being associated with slightly higher rates of nonmedical use of prescription opioids [24,26]. Therefore, prevention of initial use of opioids, establishing a timeline outlining the course and expectations for opioid use, and understanding the individual’s level of sport and specific sport involvement are all important factors that can help prevent opioid misuse.

Tapering opioid use

In addition to considering risk factors, there is a need for further research on developing guidelines for tapering opioid use in athletes. A team approach to treating chronic pain is more effective than one-to-one care, and this concept should be translated into the care of athletes [41]. An opioid risk assessment with questions about history of previous and current opioid use, other substance use, history of psychiatric disorders, and family history of substance use should also be considered [42]. Conversations should set the expectation that an athlete will have one prescriber, no early refills, and an established tapering plan [42]. Recommendations should include early tapering of opioids in athletes whose pain is not improving, a timed course of low-dose opioid analgesics as appropriate, and advising athletes to use opioid medicines sparingly [42].

Alternatives to opioids

Current evidence, as summarized in the 2022 CDC Clinical Practice Guideline for prescribing opioids for pain, supports the use of nonopioid options that may be equally or more effective than opioids in practice, particularly for acute pain conditions [43]. Nonsteroidal anti-inflammatory drugs and acetaminophen have the strongest evidence from randomized controlled trials-supported treatment evidence compared to opioids for acute musculoskeletal pain, with a better safety profile [43]. The PRICE principles (i.e., protection, rest, ice, compression, and elevation) remain the first-line treatment for acute musculoskeletal pain [44]. In the setting of pre- and postoperative pain management or initial management in the emergency department, regional anesthesia should be considered [44,45].

Moderate to strong evidence suggests that nerve blocks represent a promising approach to reducing opioid consumption in athletes by providing localized, effective pain management while avoiding systemic side effects. Studies have shown that peripheral nerve blocks, such as femoral and brachial plexus blocks, can significantly decrease the need for opioids following orthopedic surgeries or acute injuries, allowing for quicker functional recovery [44,45]. These techniques deliver targeted analgesia with reduced risk of sedation, nausea, or dependency. These benefits are particularly important for athletes aiming to maintain optimal physical and mental performance. Additionally, the use of continuous catheter-based nerve blocks can extend pain relief beyond the immediate postoperative period, further reducing opioid reliance. By integrating nerve blocks into multimodal pain management strategies, clinicians can support safer recovery pathways for athletes, prioritizing both effective analgesia and the minimization of opioid-related risks.

Cognitive behavioral therapy, another alternative to opioids, is often effective for improving functionality and well-being, targeting comorbid conditions that worsen pain perception, such as anxiety, depression, and insomnia [39,40]. Recent studies have evaluated the role of complementary and integrative health therapies, including acupuncture, massage, tai chi, and yoga, in managing pain in the general population, and these therapies should be considered for the athletic population [34,46,47].

## Conclusions

Misuse of opioids is particularly worrisome, as this may lead to more serious injuries and even death. Addressing opioid use in athletes requires a nuanced approach that balances effective pain management with the risks of dependency and performance impairment. Athletes are particularly vulnerable to opioid misuse due to their high rates of injury and pressure to maintain peak performance. Yet, studies indicate that multimodal pain management strategies can significantly mitigate these risks. If opioids are prescribed, there should be clear discussion and documentation of expectations for their use, as well as an established timeline for tapering and the introduction of lower-risk pain relief interventions and alternative therapies.

By emphasizing individualized care and adopting evidence-based protocols, medical professionals can provide safe and effective pain management for this unique population. Continued research is essential to further optimize strategies that minimize opioid use while addressing the complex physical and psychological demands of athletic recovery. This research is needed to address gaps in the literature regarding typical patterns of opioid intake in athletes, as most existing data are cross-sectional or retrospective. We recommend further prospective longitudinal studies to track opioid intake from initial exposure until retirement, while addressing relevant safety and ethical considerations. Additionally, this research should result in a clear set of guidelines for opioid prescribing, tapering, and return-to-play decision-making for distinct athletic groups. Finally, with increasing research efforts in sports medicine, it is worth considering specific populations in contact, combat, and endurance sports, given their distinct training loads, injury patterns, and prevention strategies.
